# Poly(3-hydroxybutyrate) Modified by Nanocellulose and Plasma Treatment for Packaging Applications

**DOI:** 10.3390/polym10111249

**Published:** 2018-11-11

**Authors:** Denis Mihaela Panaitescu, Eusebiu Rosini Ionita, Cristian-Andi Nicolae, Augusta Raluca Gabor, Maria Daniela Ionita, Roxana Trusca, Brindusa-Elena Lixandru, Irina Codita, Gheorghe Dinescu

**Affiliations:** 1 National Institute for Research & Development in Chemistry and Petrochemistry-ICECHIM, Polymer Department, 202 Spl. Independentei, 060021 Bucharest, Romania; cristian.nicolae@icechim-pd.ro (C.-A.N.); ralucagabor@yahoo.com (A.R.G.); 2 National Institute for Laser, Plasma and Radiation Physics, Atomistilor 409, Magurele-Bucharest, 077125 Ilfov, Romania; ionita.rosini@infim.ro (E.R.I.); daniela.ionita@infim.ro (M.D.I.); dinescug@infim.ro (G.D.); 3 Science and Engineering of Oxide Materials and Nanomaterials, University Politehnica of Bucharest, 1-7 Gh. Polizu Street, 011061 Bucharest, Romania; truscaroxana@yahoo.com; 4 “Cantacuzino” National Medical-Military Institute for Research and Development, 103 Spl. Independentei, 050096 Bucharest, Romania; brandusa_lixandru@yahoo.com (B.E.L.); icodita@cantacuzino.ro (I.C.); 5 Carol Davila University of Medicine and Pharmacy, Bulevardul Eroii Sanitari 8, 050474 Bucharest, Romania

**Keywords:** bionanocomposites, polyhydroxybutyrate, bacterial cellulose, antimicrobial activity, thermal properties, packaging, morphology

## Abstract

In this work, a new eco-friendly method for the treatment of poly(3-hydroxybutyrate) (PHB) as a candidate for food packaging applications is proposed. Poly(3-hydroxybutyrate) was modified by bacterial cellulose nanofibers (BC) using a melt compounding technique and by plasma treatment or zinc oxide (ZnO) nanoparticle plasma coating for better properties and antibacterial activity. Plasma treatment preserved the thermal stability, crystallinity and melting behavior of PHB‒BC nanocomposites, regardless of the amount of BC nanofibers. However, a remarkable increase of stiffness and strength and an increase of the antibacterial activity were noted. After the plasma treatment, the storage modulus of PHB having 2 wt % BC increases by 19% at room temperature and by 43% at 100 °C. The tensile strength increases as well by 21%. In addition, plasma treatment also inhibits the growth of *Staphylococcus aureus* and *Escherichia coli* by 44% and 63%, respectively. The ZnO plasma coating led to important changes in the thermal and mechanical behavior of PHB‒BC nanocomposite as well as in the surface structure and morphology. Strong chemical bonding of the metal nanoparticles on PHB surface following ZnO plasma coating was highlighted by infrared spectroscopy. Moreover, the presence of a continuous layer of self-aggregated ZnO nanoparticles was demonstrated by scanning electron microscopy, ZnO plasma treatment completely inhibiting growth of *Staphylococcus aureus*. A plasma-treated PHB‒BC nanocomposite is proposed as a green solution for the food packaging industry.

## 1. Introduction

Synthetic polymers have been used in packaging for many decades and at least half of their consumption is by the food packaging industry [1,2]. The increasing amount of waste from non-biodegradable plastics and the ecological problems they cause have encouraged manufacturers to find new environmentally friendly, safe and nontoxic packaging materials. Biopolymers from renewable resources are the most promising alternatives to petroleum-based polymers in food packaging [2,3]. The large application of biopolymers in this sector has huge advantages in terms of a decreased dependence on fossil fuel reserves and limitation of the environmental problems associated with synthetic polymer pollution. Polyhydroxyalkanoates (PHAs), which are obtained by bacterial synthesis, are considered as very promising candidates for food packaging and biomedical applications [4,5,6]. Their hydrolytic degradation in the environment and in living systems leads to oligomeric byproducts that are further processed by biochemical pathways [5,7]. Poly (3-hydroxybutyrate) (PHB) is the most studied of the PHAs and some of its properties are comparable to those of the petrochemical-based polymers [3]. However, shortcomings in the mechanical performance and melt processing behavior of PHB, i.e., high brittleness, poor thermal stability and difficult processing [3,8], along with insufficient barrier properties, limit its widespread use. Many attempts are being made to improve its properties for packaging application [9,10,11,12,13]. Copolyesters with higher ductility such as poly(3-hydroxybutyrate-*co*-3-hydroxyvalerate) (PHBV), poly(3-hydroxybutyrate-co-4-hydroxybutyrate) or poly(3-hydroxybutyrate-co-3-hydroxy- hexanoate) may overcome some of these shortcomings [5,9,10]. Different flexibilities and strengths may be obtained by variation of the co-monomer unit or composition in these copolyesters [10,11]. The addition of different modifiers, plasticizers, micro- and nanofillers was also tested, aiming to improve PHB properties [4,5,8,9,12]. Cellulose nanofibers are mostly used in PHB and other PHAs due to their important effect of increasing mechanical and barrier properties [1,2,3,4,6,8,13]. Bacterial cellulose nanofibers (BC) are preferred for packaging and biomedical applications due to their high purity. For example, a remarkable improvement of PHB properties was obtained by mixing PHB with medium chain length PHAs and BC as “soft” and “stiff” modifiers [8,14]. Therefore, PHB and copolymers modified by BC nanofibers are promising materials for food packaging and biomedical applications.

Several studies have shown that PHB could replace conventional plastics that are used in juices and dressings [15] or fat-rich products’ packaging [16] if its melt processability is improved. Still, the high rigidity and low flexibility of PHB leads to the cracking of jars and caps during injection molding [16]. Post-processing of PHB electrospun fiber mats by different physical treatments (annealing and cooling) resulted in PHB films with higher elongation at break and toughness compared to common compression-molded films [17]. The addition of high molecular weight natural rubber or poly(ε-caprolactone) (PCL) may also improve the elongation to break of PHB/PHBV and lead to higher thermal stability [18] or toughness [19] with the increase of rubber/PCL content in the blends. Blends of poly(lactic acid) (PLA) with 25 wt % PHB were intensively studied for food packaging applications [20,21,22]. A large amount of plasticizers must be used in these blends to ensure the high flexibility required by packaging processes [20,21,22], which is often detrimental to the mechanical properties.

However, PHB-based materials are susceptible to microbial attack, which is undesirable for food packaging applications. Different methods were proposed to obtain antimicrobial active packaging (AP) for maintaining the quality and safety of foods [23,24]. Generally, migratory and non-migratory systems were developed for this purpose [23]. In a migratory packaging system, volatile or non-volatile active components are released from the polymer matrix to the surface of the food and, in the case of non-migratory systems, the antimicrobial agents or oxygen/moisture absorbers are bound to the polymer packaging.

Several agents were tested to obtain PHB-based biomaterials with antimicrobial activity for AP [25,26,27,28,29,30,31]. Natural antimicrobial agents such as vanillin [25], sophorolipid [26] or eugenol [27] were incorporated in PHB in different proportions using a solvent casting-evaporation method with chloroform as a solvent. Vanillin and eugenol in small concentrations (80 μg/g PHB) [25,27], and sophorolipid in a much higher concentration (9–29 wt %) [26] were found to be effective against a large number of bacterial species. Carvacrol, a constituent of several essential oils, was also incorporated in PLA/PHB blends and improved their antioxidant and antibacterial activity [27]. However, the incorporation of these natural antimicrobial agents in PHB frequently leads to the decrease of thermal stability and mechanical properties [25,28].

Synthetic antibacterial agents were also tested in PHB formulations; however, their concentration should be limited in the case of food packaging application [29,30]. Triclosan showed good bactericidal activity against *Escherichia coli* and *Staphylococcus aureus* after its incorporation in PHBV, and increased its flexibility, but significantly reduced its elastic modulus and tensile strength [29]. PHB modified by chlorhexidine digluconate showed a controlled release of the antibacterial agent into a food area [30]. Similarly, PHB/PCL/organo-clay nanocomposites activated with nisin, an antibacterial peptide, showed good inhibiting effect against *Lactobacillus plantarum* and extended the shelf life of processed meat [31].

In recent years, metal nanoparticles have been intensively studied for their ability to induce antimicrobial activity in PHB films for AP applications [32,33,34,35,36,37,38]. For example, zinc oxide (ZnO) nanoparticles, which are efficient UV blockers, were incorporated in PHB using a solution casting technique [32]. The nanocomposite films showed good antibacterial activity against both Gram-positive and Gram-negative bacteria, depending on the ZnO concentration within the nanocomposite. Furthermore, PHB/ZnO films showed better mechanical properties and thermal stability compared to the PHB matrix [32]. Similar improvement was reported for (3-hydroxybutyrate-co-3-hydroxyvalerate) (PHBV) reinforced with ZnO [33]. An optimal concentration of 4.0 wt % ZnO determined the maximum tensile strength, Young’s and storage moduli, crystallinity and barrier properties, and antibacterial activity. Another route to obtain PHB films with antimicrobial activity for AP was proposed by Castro-Mayorga et al. [34]. They obtained PHB nanocomposites containing silver nanoparticles using a biological synthesis without the addition of a reducing agent to prepare these nanoparticles. The nanocomposite films showed strong antimicrobial activity against *Salmonella enteric* and *Listeria monocytogenes*, known food-borne pathogens. Moreover, no significant change of thermal stability or biodegradability of PHB was caused by these nanoparticles [34]. Another “green” method to obtain silver nanoparticles and PHB‒Ag nanocomposites consists of the grafting of PHB onto the chitosan biguanidine, which reduced silver nitrate to silver nanoparticles [35]. These nanocomposites showed good antibacterial activity against Gram-positive and Gram-negative bacteria. PHBV films coated with PHBV/ZnO ultrathin fiber mats and annealed at 160 °C showed effective and prolonged antibacterial activity against *Listeria monocytogenes* [36].

In most of the methods proposed so far, large amounts of chloroform or other solvents are used to prepare the PHB films with antimicrobial activity, which may cause environmental or health problems. Melt processing is environmentally friendly and cost-effective and, thus, a preferred choice for industrial scale-up. Moreover, the incorporation of metal nanoparticles in the whole PHB matrix leads to the use of a large amount of nanoparticles for the same effect, with only the surface ones being efficient as antibacterial agents. Moreover, the migration of nanoparticles from the bulk to the surface is a difficult process in PHB due to its high crystallinity and additional additives like lubricants, plasticizers, surfactants being necessary to increase migration. Surface treatment of PHB melt-processed films using plasma is an innovative and environmentally friendly approach to obtain antibacterial activity with a minimal change in bulk properties.

In this work, PHB was modified with different amounts of BC nanofibers as a reinforcing agent [4]; PHB‒BC nanocomposite films were produced by melt compounding and compression molding and were plasma-treated to improve their properties and enhance antibacterial activity. Moreover, a PHB–BC–ZnO nanocomposite film was prepared using a new plasma coating process. ZnO nanoparticles were selected as an affordable and safe solution for food packaging, ZnO being recognized as safe by the U.S. Food and Drug Administration [36]. Earlier works have reported on the effect of low-pressure plasma treatment in the modification of PHB by grafting, polymerization or functionalization for biomedical applications [39,40,41,42,43]. Low-pressure plasma treatments were mostly used to improve the surface hydrophilicity of PHB/PHBV films for enhancing cell compatibility [44,45,46,47,48,49,50,51,52]. The treatments were applied to increase cell adhesion and proliferation for scaffolding and other biomedical purposes. In a study, PHB was plasma-activated and subsequently silver-coated for being used in packaging [52].

However, low-pressure plasma processes have limitations due to the complicated installations and high costs inherent to vacuum technology. Moreover, the influence of plasma treatment on PHB‒nanocellulose composites was not yet studied. In this work we propose a simple atmospheric pressure plasma treatment and coating process, which ensure the surface modification of PHB and ZnO nanoparticles deposition. The effect of the plasma treatment and ZnO plasma coating on the thermal, mechanical and antimicrobial properties of PHB and its nanocomposites with 2 and 5 wt % bacterial cellulose nanofibers was investigated here for the first time. Plasma treatments modified to a different extent the thermal and mechanical properties of PHB and its antibacterial activity depending on composition and treatment conditions. Plasma-treated PHB‒BC nanocomposites are new materials that are proposed as a “green” solution for food packaging. This work is an important step for the design of new biomaterials obtained by eco-friendly processes, as alternatives to non-biodegradable plastics commonly used in the food packaging industry.

## 2. Materials and Methods

### 2.1. Materials

Bacterial cellulose pellicles consist of a network of entangled cellulose nanofibers, with a width of 40–110 nm [53]. Individual bacterial cellulose nanofibers were produced by the mechanical disintegration of bacterial cellulose pellicles using a high-speed blender for 30 min and a vertical colloid mill at about 2000 min^−1^ for 180 min. Cold water (5–10 °C) was added to prevent temperature rising above 40 °C. BC nanofibers with widths mostly between 50 and 90 nm and a thickness of 8–10 nm, as determined by atomic force microscopy (AFM, JPK Instruments, Berlin, Germany), peak force quantitative nanomechanical mapping (QNM) mode, were obtained by defibrillation. The suspension containing BC nanofibers was concentrated in a rotary evaporator (Heidolph Instruments, Schwabach, Germany) at 40 °C and then freeze-dried using FreeZone 2.5 L (Labconco, Kansas City, MO, USA). Pelletized PHB from Goodfellow (Huntingdon, UK) with a density of 1.25 g cm^−3^ was used to prepare the nanocomposites. A rough proportion of 10% tributyl citrate (TBC) plasticizer was estimated in the commercial PHB granules [12]. The argon (Ar) gas used to generate plasma was 99.999% purity. The ZnO nanoparticles with a mean diameter of 50 nm and ethanol with a purity higher than 99.8% were purchased from Sigma-Aldrich (St. Louis, MI, USA) and used as received.

### 2.2. Preparation of PHB Nanocomposite Films

PHB pellets and BC were dried in vacuum ovens at 60 °C for 2 h and 50 °C for 4 h. Cellulose nanofibers (2 and 5 wt %) were added in melted PHB using a Brabender LabStation (Duisburg, Germany) with a mixing chamber of 30 cm^3^. Melt mixing was carried out at 170 °C for 8 min starting from the melting of PHB at a rotor speed of 40 min^−1^. Films with a thickness of 200 µm were obtained by compression molding using an electrically heated press (P200E, Dr. Collin, Ebersberg, Germany) at 175 °C, with 120 s preheating (5 bar), 75 s under pressure (100 bar), and cooling for 1 min in a cooling cassette.

### 2.3. Plasma Treatments of PHB Nanocomposite Films

**Figure 1:**
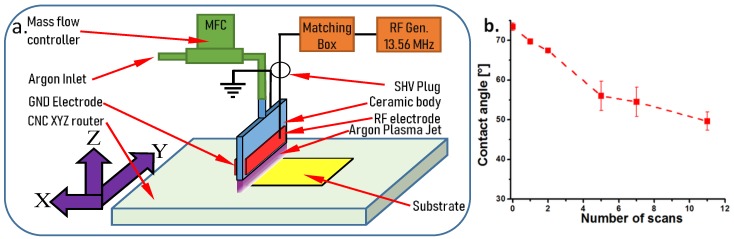
Experimental setup for the DBD plasma treatment of PHB nanocomposite films (substrate) (**a**); contact angle of the nanocomposite films vs. the number of scans (**b**).

The experimental system designed for the dielectric barrier discharge (DBD) plasma treatment of PHB nanocomposite films is shown in Figure 1a. In the first setup, the nanocomposite film was placed on a CNC XYZ router table (StepCraft 300, Stepcraft, Iserlohn, Germany) and swept by a longitudinal jet of atmospheric pressure Ar plasma. The distance between the polymer film and the longitudinal nozzle was 1.8 mm and the length and thickness of the plasma jet were 10 cm and 1 mm, respectively. The plasma source consists of a flat ceramic body that allows the gas flow and two metal electrodes, one connected to the ground (GND) and the other to the radiofrequency (RF) generator (Cesar 136 13.56 MHz—Advanced Energy Industries, Metzingen, Germany). The discharge impedance was matched to the generator via an automatic impedance matching network (matching box Advanced Energy Navio). The Ar feeding through the top of the plasma source was assured by a mass flow controller (EL-FLOW 10 slm—Bronkhorst, AK Ruurlo, The Netherlands).

The number of scans was determined based on contact angle measurements. Different number of scans, from 1 to 11, was carried out with the DBD plasma source on the surface of PHB nanocomposites and the contact angle was measured with a CAM 200 (KSV Instruments, Helsinki Finland) equipped with a high resolution camera (Basler A602f, Basler, Ahrensburg, Germany) and an auto-dispenser, at room temperature and ambient humidity. Five scans was considered optimal (Figure 1b). All the plasma-treated nanocomposites were denoted with a “p” after their name. In the second setup, the nanocomposite films were plasma-treated in the same conditions as mentioned in the first setup for increasing their hydrophilicity and then coated with ZnO nanoparticles dispersed in an alcohol solution using an ultrasonic spraying device. In both experimental setups, the control of the devices involved in the experiments was carried out by a computer using our own software.

### 2.4. Characterization 

#### 2.4.1. Scanning Electron Microscopy Coupled with Energy-Dispersive X-ray Analysis (SEM-EDX) 

SEM images were captured with a Quanta Inspect F scanning electron microscope (FEI-Philips, Hillsboro, OR, USA), equipped with a field emission gun, working at an accelerating voltage of 30 kV with a resolution of 1.2 nm. The surface composition was analyzed with an energy dispersive X-ray (EDX) spectrometer coupled to SEM, with a resolution of 133 eV at MnKα. The blends and nanocomposites films were sputter-coated with gold before analysis.

#### 2.4.2. Thermal Characterization

Thermogravimetric analysis (TGA) was used to characterize the thermal stability of PHB nanocomposites before and after treatments. TGA was carried out on TA-Q5000 V3.13 system (TA Instruments Inc., New Castle, DE, USA) with signal resolution of 0.01 µg, using nitrogen as the purge gas at a flow rate of 40 mL/min. Samples were heated from 25 °C to 700 °C at a heating rate of 10 °C/min.

Differential scanning calorimetry (DSC) was carried out with a DSC Q2000 from TA Instruments (New Castle, DE, USA) under a helium flow (100 mL/min). Samples weighing around 8 mg were cooled to −65 °C, allowed to stabilize for several minutes, heated to 200 °C and held at that temperature for 3 min to delete the thermal history, then cooled to −65 °C, kept isothermal at that temperature for 3 min and heated again to 200 °C at a constant heating/cooling rate of 10 °C/min. The instrument was calibrated using indium standard (TA Instruments) and has a temperature precision of ±0.01 °C and a calorimetric precision of ±0.05%. The melting temperature (*T*_m_) was taken as the peak temperature of the melting endotherm. The degree of crystallinity (*C*) was calculated from the second melting scan as in Equation (1):

(1) $$C=\frac{\Delta H}{\Delta H_{0}\;w_{PHB}}\;\cdot \;100$$

where Δ*H*_m_ and Δ*H*_0_ are the apparent melting enthalpy of the nanocomposite and of 100% crystalline PHB, respectively, while *w*_PHB_ is the weight fraction of PHB in nanocomposite. Δ*H*_0_ was taken as 146 J/g [32].

#### 2.4.3. Dynamic Mechanical Analysis (DMA)

The thermomechanical properties of blends and nanocomposites were analyzed using a DMA Q800 (TA Instruments, New Castle, DE, USA), which has a force resolution of 0.00001 N and a strain resolution of 1 nm, operating in multi-frequency–strain mode. The films were maintained at room temperature for four weeks before characterization. The bar specimens with the length × width of 20 mm × 6 mm were obtained by cutting. Samples in duplicate were cooled to −15 °C with 10 °C/min, equilibrated for 5 min at this temperature and heated to 160 °C with a heating rate of 3 °C/min.

#### 2.4.4. Fourier Transform Infrared Spectroscopy (FTIR)

FTIR spectrometer with ATR setup (Tensor 37) from Bruker Optics (Ettlingen, Germany) was used for the analysis of plasma-treated nanocomposite films. The spectra were collected at room temperature, from 4000 to 400 cm^−1^, at a resolution of 4 cm^−1^ using 16 scans. Spectra were baseline-corrected using the OPUS software (Bruker Optics, Ettlingen, Germany).

#### 2.4.5. Peak Force Quantitative Nanomechanical Mapping 

AFM images of PHB and nanocomposite with 5 wt % BC (PHB‒5BC) were captured using a MultiMode 8 AFM instrument (Bruker, Santa Barbara, CA, USA) in peak force QNM mode. Measurements were carried out at room temperature with a scan rate of 0.7–0.8 Hz and a scan angle of 90° using an etched silicon tip (nominal radius 8–10 nm) with a cantilever length of 225 μm and a resonance frequency of about 75 kHz. The data and images were processed with NanoScope software version 1.20.

#### 2.4.6. Tensile Characterization

The tensile properties of PHB and PHB‒BC nanocomposites were measured at room temperature using an Instron 3382 universal testing machine (Instron, Norwood, MA, USA) with a crosshead speed of 2 mm/min. Five specimens according to ISO 527 were tested for each nanocomposite and the results were analyzed using the Bluehill 2 software (Instron, Norwood, MA, USA).

#### 2.4.7. Antibacterial Activity

The antibacterial activity of plasma-treated nanocomposite films was evaluated against *Staphylococcus aureus* by the colony counting method [54]. Square shaped fragments of 5 × 5 mm^2^ were cut from the nanocomposite films and were placed in sterile Petri dishes. The plasma-treated samples were placed with the impregnated face up. The Petri dishes with the nanocomposite fragments were exposed to a UV source for 15 min. Ten 10-mm square fragments of reference and nanocomposite films were placed in a sterile 25/25 mm tube. An aliquot of 1 mL bacterial culture (*Staphylococcus aureus* ATCC 6538 or *Escherichia coli* ATCC 35218) was added over the nanocomposite fragments to a concentration of about 10^2^ UFC / mL. The tubes were then incubated with stirring at 36 ± 1 °C for 24 h. A set of 10 fragments from the untreated reference was seeded with 1 mL of non-inoculated nutrient broth and served as a sterility control. The concentration of the bacterial suspensions used in the experiment was checked by inoculating 50 μL of each suspension, in triplicate, onto a blood agar plate. After incubation, 25 mL of physiological saline solution was added to each tube, the tubes were vortexed and then 100 μL were transferred into Corning tubes with 9.9 mL of physiological saline solution, diluted at 10^−2^ and then seeded on blood agar plates. After the incubation of seeded plates, the colonies were counted for each of the analyzed samples. The percentage reduction in bacterial growth, X (%) was calculated as follows [55]:

X (%) = (viable count at 0 h − viable count at 24 h) × 100/viable count at 0 h(2)

## 3. Results and Discussion

### 3.1. Thermogravimetric Analysis

Plasma surface treatment may affect the thermal stability of PHB nanocomposites films due to the reactive species, radicals, excited atoms or charged particles that are generated during this treatment. Therefore, the influence of the plasma treatment on the thermal behavior of PHB modified with cellulose nanofibers was investigated by thermogravimetric analysis.

**Figure 2:**
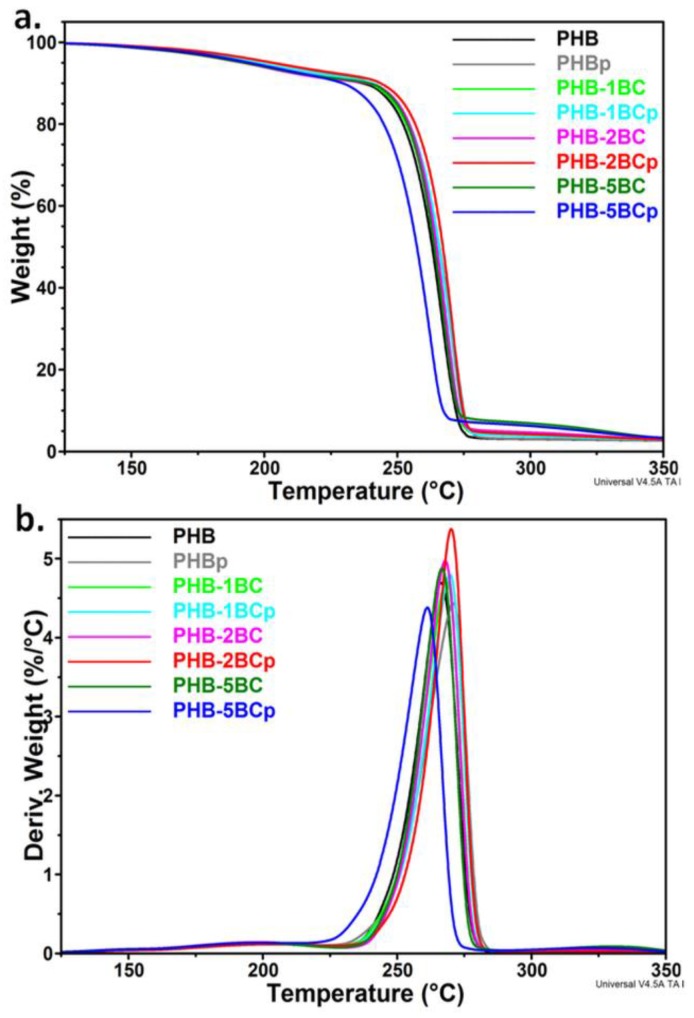
TGA (**a**) and DTG (**b**) curves of PHB nanocomposites with different BC content before and after the plasma treatment.

The thermal degradation of PHB and nanocomposites consists in a main degradation step between 230 °C and 280 °C (Figure 2a). Two small shoulders were observed in the derivative thermogravimetric (DTG) curves (Figure 2b), one at about 195 °C, probably determined by the release of TBC plasticizer from the PHB matrix [12], and the second at about 330 °C. This second shoulder is absent in PHB films and occurs only in nanocomposites, increasing with the amount of BC (see Figure S1). Therefore, it may be attributed to cellulose decomposition.

The thermal stability is slightly influenced by the plasma treatment or composition (Figure 2). An increase in the maximum degradation temperature (*T*_max_) of 1.5–4.4 °C was observed following plasma exposure for pristine PHB and nanocomposites with 1–2 wt % BC; however, a decrease of 5.2 °C was noticed for PHB‒5BC (Table 1). This suggests a higher plasma sensitivity of cellulose, also noticed in our previous works [56,57]. The differences in the onset degradation temperature (*T*_on_) values before and after the plasma treatment were similar to those of *T*_max_. However, *T*_5%_ (temperature at 5% weight loss) values showed different changes following the treatment (Table 1). This characteristic temperature may be considered as a measure of the thermal stability during melt processing, being close to the melt temperature range. An important increase of the *T*_5%_ value, more than 10 °C, was observed for PHB and PHB‒2BC nanocomposite after the plasma treatment. This may be due to the chemical changes of PHB induced by the plasma exposure.

**Figure 3:**
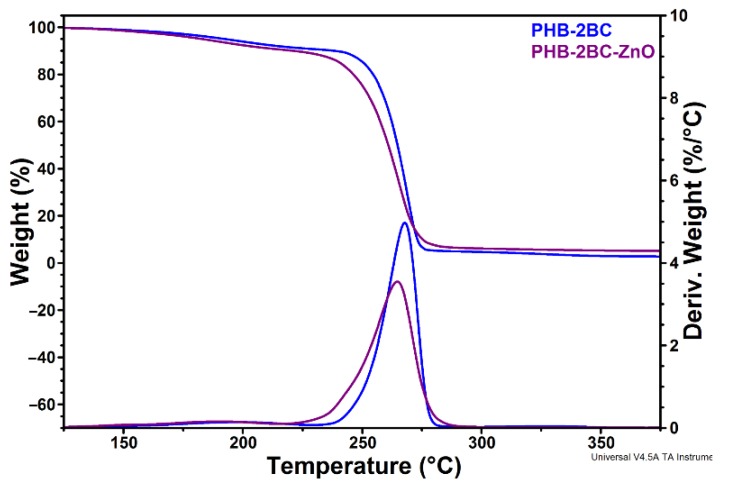
TGA-DTG curves of PHB nanocomposite plasma-coated with ZnO nanoparticles.

Plasma deposition of ZnO nanoparticles decreased the *T*_max_ of PHB‒2BC nanocomposite by 2.9 °C. Similarly, the *T*_on_ of the nanocomposite decreased by about 5 °C (Figure 3, Table 1).

A decrease of the *T*_max_ of about 12 °C was observed after the incorporation of ZnO nanoparticles (6 wt %) in PHBV using a melt mixing procedure [36]. However, a strong increase of the thermal stability, with about 20 °C, was reported for PHB and PHBV nanocomposites with only 2 wt % ZnO prepared by solution casting [32,33]. The different effect of ZnO nanoparticles on the thermal stability of PHAs may be due to the different procedure or degree of dispersion and other factors [58]. However, the slight decrease in thermal stability observed for ZnO plasma-coated PHB may be rather an effect of the plasma treatment and of the active species that appear during this treatment. Nanocomposite films were analyzed by ATR-FTIR to observe the surface changes induced by the treatments.

### 3.2. FTIR Analysis

**Figure 4:**
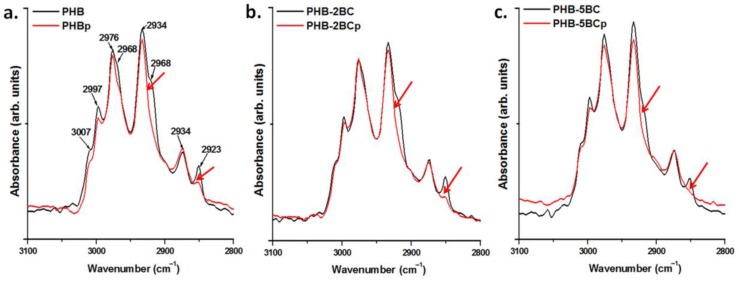
FTIR spectra of untreated and plasma-treated PHB and nanocomposites in the C–H stretching vibration region.

FTIR spectra of PHB and PHB nanocomposites with 2 wt % and 5 wt % BC are given in Figure S2. The addition of BC in PHB leads to no spectral differences because of the small amount of cellulose. Several differences were observed after the plasma treatment of PHB and nanocomposites (Figure S3). Most of the differences occur in the range from 3100 to 2800 cm^−1^, assigned to C‒H stretching modes (Figure 4).

The bands in the range 3015–2955 cm^−1^ are assigned to CH_3_ asymmetric stretching vibrations and those in the range 2940–2915 cm^−1^ to CH_2_ asymmetric stretching vibrations [59]. The range from 2885 to 2845 cm^−1^ is characteristic of symmetric stretching modes of CH_3_ and CH_2_ [59,60]. The peaks at 2976 cm^−1^, 2934 cm^−1^ and 2874 cm^−1^ and the shoulder at 2923 cm^−1^ arise from the crystalline state and that at 2997 cm^−1^ from the amorphous state [61,62]. The shoulder at 3007 cm^−1^, related to CH_3_ asymmetric stretching, indicates the presence of intermolecular CH…O hydrogen bonds in PHB crystals [61]. Assignments of the peaks in Figure 4 (3050–2800 cm^−1^ region) are given in Table S1. PHB and nanocomposites show similar response to the plasma treatment, i.e., disappearance of the shoulder at 2923 cm^−1^ and of the peak at 2851 cm^−1^. Previous works have shown that the two peaks at 2934 and 2923 cm^−1^, which are related to the CH_2_ asymmetric stretching [62], result from the crystal field splitting, caused by inter- or intramolecular interactions [61]. Therefore, the disappearance of the peak at 2923 cm^−1^ may be related to changes in the molecular packing and interactions caused by the plasma treatment. The same reasons may cause the reduction or disappearance of the peak at 2851 cm^−1^, which is attributed to the symmetric CH_2_ stretching vibrations. The cleavage of chemical bonds and release of small molecular products and the occurring of cross-linking reactions may also be reflected in this FTIR region. The disappearance of several peaks in the range from 800 to 400 cm^−1^ was also observed in all the samples after the plasma treatment and it may be explained by the cleaning effect of Ar plasma [56,57].

**Figure 5:**
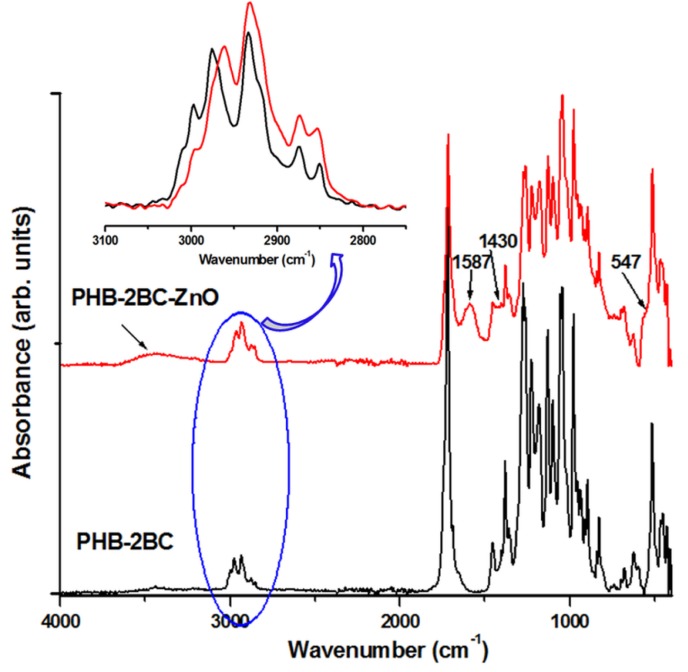
FTIR spectra of PHB‒2BC and ZnO plasma-coated nanocomposite.

In contrast, a significant effect of ZnO plasma coating on PHB nanocomposite film with 2 wt % BC was observed (Figure 5). ATR-FTIR is surface-sensitive and it is a very suitable method to investigate the effects of plasma coating with ZnO nanoparticles. The main changes in the FTIR spectra are summarized below:

(i) the appearance of a new wide band at 3600–3100 cm^−1^, which is ascribed to free and hydrogen bounded OH groups in alcohol and water; this probably comes from the alcoholic suspension of ZnO nanoparticles used for the treatment;

(ii) the shift of the peak ascribed to CH_3_ asymmetric stretching vibrations, from 2976 to 2961cm^−1^; this shift may be caused by several factors; however, the participation of methyl groups on the surface of PHB in reactions with the active species from ethanol and ZnO under the plasma bombardment is, probably, the most important [63]; the catalytic effect of Zn should have a remarkable enhancing role; moreover the absorption of ethanol, acetaldehyde, ethylene, water or other reaction products could also take place;

(iii) the appearance of a new large peak at 1587 cm^−1^, which may be due to the C=C stretching vibrations of polymer‒ZnO complex ion, similar to other observations [64]; ethylene active species result from ethanol under plasma and catalytic effect of Zn [63];

(iv) the appearance of a new band at 1430 cm^−1^, in the range characteristic to the CH_3_ and CH_2_ deformation vibrations, which may be ascribed to the metal complexes; similarly, the asymmetric and symmetric stretching vibrations of metal carboxylate complexes occur at 1590 and 1430 cm^−1^ [65].

(v) the appearance of a shoulder at 547 cm^−1^, which is characteristic to ZnO nanoparticles [66].

Overall, the peaks observed in the FTIR spectra of PHB nanocomposite after ZnO coating support the formation of polymer‒ZnO complex species, i.e., strong chemical bonding of the metal nanoparticles on the surface of PHB nanocomposite. The role of cellulose nanofillers in the bonding of Zn nanoparticles could be also an active one.

### 3.3. Differential Scanning Calorimetry

**Figure 6:**
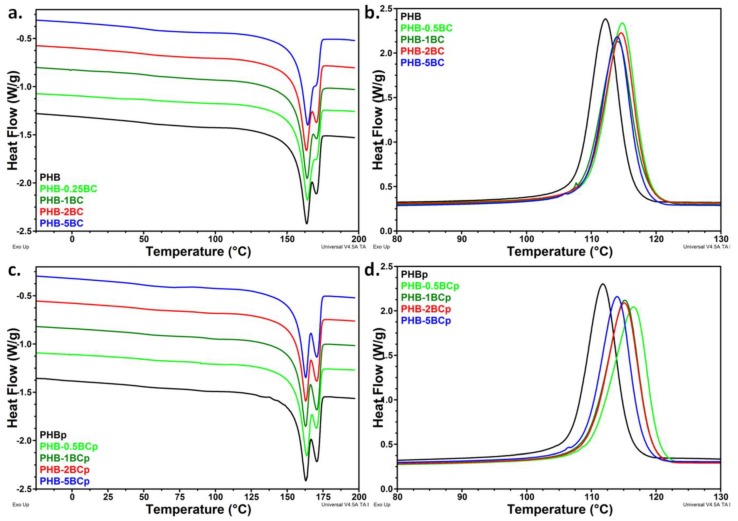
DSC melting (**a**) and cooling (**b**) scans of PHB nanocomposites; DSC melting (**c**) and cooling (**d**) scans of PHB nanocomposites after the plasma treatment.

DSC analysis may highlight the changes induced by the plasma treatments in the crystalline and amorphous phases. The heating and cooling DSC thermograms of nanocomposites before and after the plasma treatment are shown in Figure 6a–d. PHB shows double melting peaks due to melting, re-crystallization and re-melting during heating with the higher temperature peak arising from the re-crystallized polymer [67]. Different lamellar thickness and perfection may be another cause of the double melting peak besides the different molecular weight species and orientations [12]. Moreover, much attention has been paid to the influence of the compositional heterogeneity as a source of the multiple melting behavior of PHB compositions [8,68].

Minor changes in the melting temperature and enthalpy of nanocomposites containing cellulose nanofibers vs. pristine PHB were observed (Table 2), in agreement with other reports [69,70]. In particular, the addition of 2–10% cellulose crystals in PHB did not change its melting temperature or crystallinity [70]. However, all the nanocomposites showed higher crystallization temperature (*T*_c_) compared to pristine PHB (Table 2), which is a result of the nucleating effect of BC [71].

Furthermore, the melting temperature of PHB and nanocomposites did not change significantly after the plasma treatments. However, a slight but systematic increase of *T_c_* (by 1–2 °C) and decrease of crystallinity (with less than 2%) were observed after the plasma treatment of nanocomposites with less than 5 wt % BC (Figure 6b,d; Table 2). In general, the changes in the bulk properties following the plasma treatments are small due to the surface character of these treatments. The transformations induced by plasma in polymers occur in a small depth from the surface, keeping unmodified the bulk material and, therefore, its properties [72]. The slight increase of *T_c_* and decrease of crystallinity after plasma exposure may be caused by several factors: (i) the slight increase of temperature at the surface of nanocomposites, which induces faster aging of PHB [71]; (ii) new active species and functions generated by plasma, which act as nucleating sites; and (iii) crosslinking reactions involving surface PHB chains.

**Figure 7:**
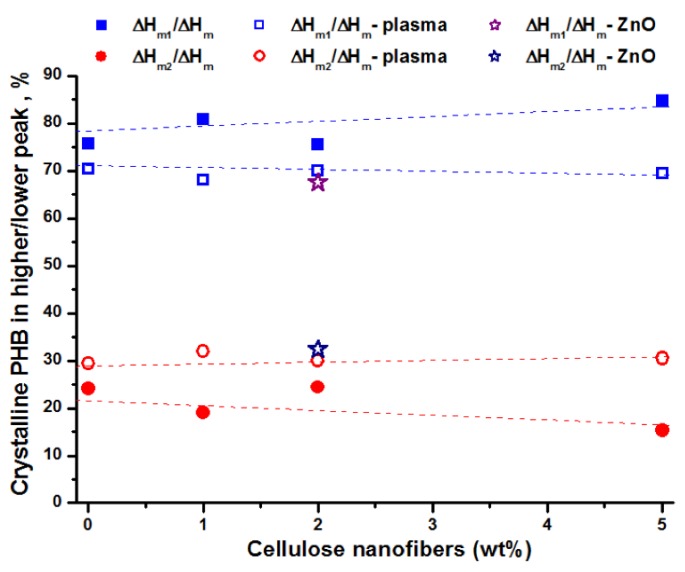
Influence of cellulose nanofibers concentration and plasma treatments on the proportion of crystalline PHB with lower (upper squares) and higher melting temperature (lower circles).

An important aspect observed after the plasma treatment is the increased proportion of crystalline PHB with higher melting temperature (higher melting peak), coming from recrystallization. The proportion of crystalline PHB corresponding to the higher and lower melting peaks was calculated by dividing the enthalpy of the peak to the total enthalpy (Δ*H*_m1_/Δ*H*_m_ and Δ*H*_m2_/Δ*H*_m_) (Figure 7).

Before the plasma treatment, a decreasing tendency of Δ*H*_m2_/Δ*H*_m_ proportion with the concentration of cellulose nanofibers was observed and almost no variation after plasma curing. Therefore, it may be assumed that cellulose hinders the recrystallization; however the plasma treatment favors this process. Thus, plasma bombardment increased with a couple degrees the local temperature and the polymer chains mobility is also increased favoring the formation of larger, more stable crystals during melting. Moreover, the small amount of new functions induced by plasma may act as seeds for the melting recrystallization process. Higher surface crystallinity was observed after oxygen and carbon dioxide vacuum plasma treatment of PHB, which was explained by the preferential etching of the softer amorphous parts on the surface of PHB in plasma treatment [51].

These effects are more pronounced in the case of ZnO plasma-coated PHB‒2BC nanocomposite (Figure S4): higher crystallinity and higher proportion of more perfect crystallites. ZnO acts probably as a nucleating agent enhancing the crystallization. Previous works have shown the effectiveness of ZnO nanoparticles as nucleating agents, raising the crystallinity of PHB or PHBV in PHAs-ZnO nanocomposites [32,33].

### 3.4. Dynamic Mechanical Analysis

**Figure 8:**
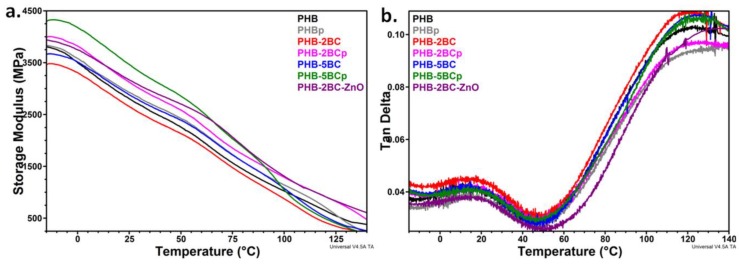
Storage Module (**a**) and tangent of mechanical loss angle (loss factor, tan δ) (**b**) of PHB nanocomposites with 2 and 5 wt % BC before (-) and after plasma treatment (p).

The glass transition temperature (*T*_g_) cannot be detected in the DSC diagrams (Figure 6). Moreover, the mechanical properties might be modified by the plasma treatment [41,51]. Therefore, dynamic mechanical analysis (DMA) was used to characterize the viscoelastic behavior and transitions (Figure 8). The values of the *T*_g_ and storage modulus (*E’*) at different temperatures were collected in Table 3.

The plasma treatment has as result a slight increase of the storage modulus of PHB, between 0.7% and 11% depending on the temperature; however, a strong increase of the storage modulus was observed for nanocomposites after the plasma treatment. In particular, an increase between 15% and 43% was noticed in the case of PHB‒2BC and an increase of 17–20% for PHB‒5BC. ZnO plasma coating led to a further increase in the storage modulus of PHB‒2BC of 21–46%. Data available in the literature on the influence of plasma treatment on PHA mechanical properties are scarce [41,51]. PHB films modified on their surface with poly(acrylic acid) using a vacuum plasma process showed increased tensile strength and Young’s modulus indicating recrystallization during plasma irradiation [41]. Small change of the storage modulus was also reported in the case of O_2_ plasma-treated PHB [51].

Correlating DMA, DSC and FTIR results, it can be assumed that the plasma treatment led to both physical (different crystal thicknesses and perfection, accelerated aging, heterogeneous nucleation) and chemical changes (active species, functionalization and/or crosslinking) that decrease the chain mobility in the surface layers and increase the stiffness, leading to this remarkable increase of the storage modulus. The dynamic mechanical properties are also sensitive to the concentration of cellulose in nanocomposites, an increase of the storage modulus with the increase of BC concentration being noticed after the treatment. The largest increase of stiffness (27%) compared to pristine PHB was observed for the nanocomposites with 5 wt % BC. The highest increase of stiffness at 100 °C was noticed in the case of PHB‒2BCp and PHB‒2BC‒ZnO, with 26% and 28%, showing a synergistic effect of BC and plasma on the mechanical properties of PHB. It can be assumed that the plasma modification of nanocellulose is easier compared to PHB due to its more polar character; however, the addition of cellulose nanofibers may also induce a higher porosity, which may favor the diffusion of the active species generated during the plasma treatment. To elucidate this aspect, plasma-treated films were investigated by SEM.

Two relaxations were observed in the tan δ versus temperature curves, the first at 14…18 °C, depending on the treatment and composition, which corresponds to the glass transition of PHB and the second at 122…136 °C, which is a transition associated to the slippage between crystallites [8,73] A slight increase of *T*_g_, with 1–3 °C, was noticed for the plasma-treated films, showing a restriction of PHB chain mobility in the amorphous regions induced by plasma. Besides the influence of the larger crystals on the surrounding amorphous phase, the chemical changes and, especially crosslinking, may decrease the chain mobility leading to higher *T*_g_ values after the treatments. These changes are supported by increased values of the *T*_α_* transition after the plasma treatment for all the samples with about 3 °C, showing a more difficult slippage between crystallites after the treatment. The highest *T*_α_* (136 °C) was noticed for ZnO plasma-coated PHB‒2BC nanocomposite, showing a high influence of ZnO plasma treatment; it can be assumed that ZnO nanoparticles are strongly bond to the surface of the films, which is consistent with the FTIR results.

### 3.5. Tensile Characterization

**Figure 9:**
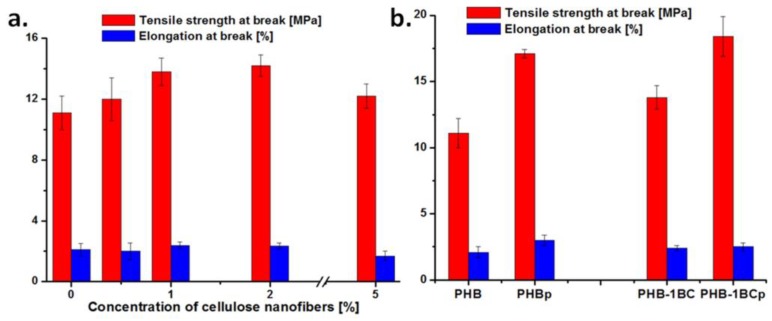
Tensile tests results for (**a**) nanocomposites with different amount of cellulose nanofibers and (**b**) PHB and PHB‒1BC before and after plasma treatment.

Tensile tests were carried out to determine the influence of cellulose nanofibers on the strength and flexibility of the prepared films (Figure 9a). An increase in the tensile strength of nanocomposites with BC concentration was observed, although no chemical modification was applied, and no significant variation of the elongation at break. Thus, an increase of tensile strength by 24% and 28%, from 11.1 MPa for PHB to 13.8 MPa for the nanocomposite with 1 wt % BC and 14.2 MPa for the nanocomposite with 2 wt % BC was noticed. A concentration of 5 wt % BC nanofibers led to a less significant increase in tensile strength. Similar results were reported for PHBV with 12 mol % hydroxyvalerate (HV) reinforced with cellulose nanowhiskers (CNW), with 2.3 wt % the optimum concentration of CNW [74]; however, no variation in mechanical properties was observed in the case of PHBV (low and high HV content) incorporating bacterial cellulose nanowhiskers [75], both obtained using a solution casting technique. On the other hand, a slight increase of tensile strength was reported for PHBV nanocomposites with nanofibrillated cellulose prepared by melt mixing [76]. Therefore, the increase in tensile strength (Figure 9a) may be caused by stronger interfacial bonding between PHB and BC nanofibers, favored by melt compounding, and by a good dispersion of nanofibers. The leveling off observed for 5 wt % BC may be attributed to the PHB embrittlement caused by BC nanofiber agglomeration. The most favorable mechanical behavior in terms of improvement of properties vs. concentration of nanofiller was observed for the nanocomposite with 1 wt % BC. This was also characterized after treatment with plasma.

A remarkable increase of tensile strength was observed after the plasma treatment for both PHB and the PHB‒1BC nanocomposite, with 54% and 21%, respectively. This is in line with the important increase of storage modulus observed after the plasma treatment (Figure 8).

### 3.6. Morphological Analysis by SEM and AFM

**Figure 10:**
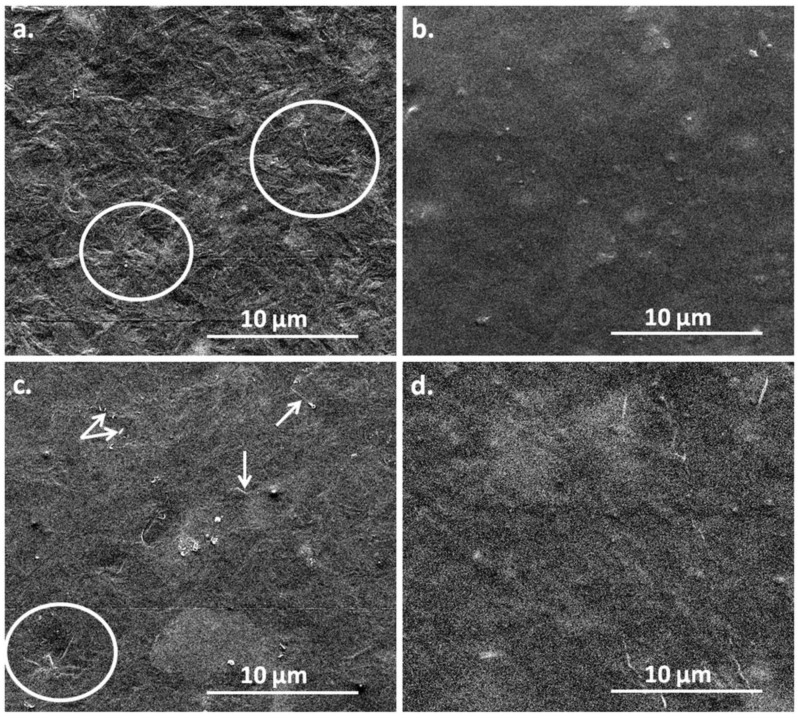
SEM images of PHB and PHB‒5BC before (**a**,**c**) and after (**b**,**d**) the plasma treatment.

The SEM image of the PHB film (Figure 10a) shows an ordered spherulitic structure (circled areas), which is less obvious after the plasma treatment (Figure 10b).

This image was captured one week after the plasma treatment of PHB; however, an embossed surface was observed 18 h after the treatment (Figure S5). This pattern is similar to an “egg box” structure. The embossing of the film surface may be due to the different effect of the plasma exposure on the crystalline and amorphous regions in PHB. The areas with higher crystallinity or the spherulites are more resistant to plasma exposure than the amorphous ones, leading to different degree of deformation. It is worth to mention that several particles mostly in nanometric range were observed on the surface of PHB and nanocomposite, before and after the treatment. They may be inorganic nanoparticles usually found in commercial PHB samples.

The spherulitic morphology is hardly seen on the surface of PHB‒5BC nanocomposite (Figure 10c) because it is masked on certain areas by the presence of cellulose nanofibers. They are well dispersed on the surface of nanocomposite and they have a thickness of less than 100 nm and lengths from 500 nm to several microns. Similarly with PHB, a ordered structure was not observed on the surface of PHB‒5BC nanocomposite after the plasma treatment.

**Figure 11:**
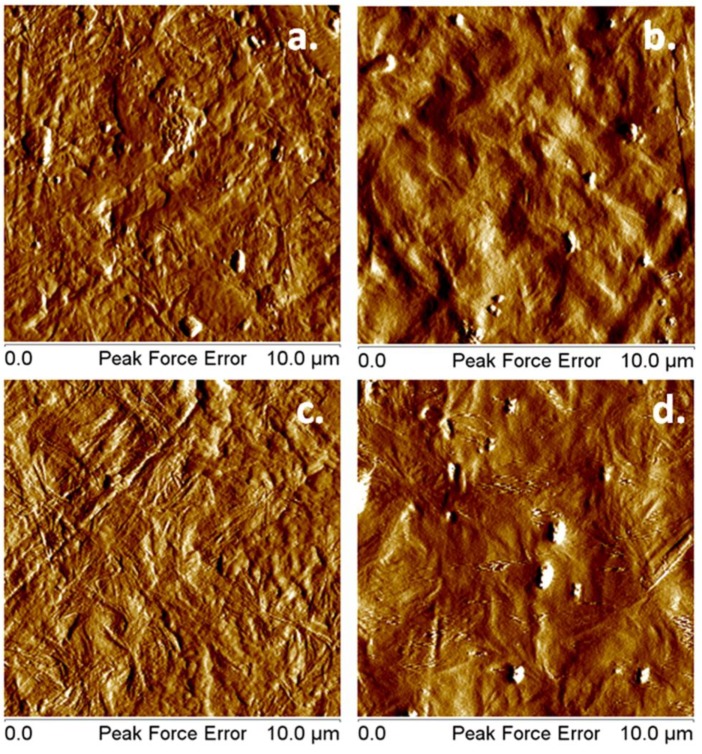
AFM peak force error images of PHB and PHB‒5BC before (**a**,**c**) and after the plasma treatment (**b**,**d**).

Atomic force microscopy is a sensitive tool to analyze nanostructures. Figure 11 shows that the organized structure observed in PHB and PHB‒5BC was maintained after the plasma treatment, but was attenuated.

The lamellar morphology of PHB may be seen in Figure 11a and, especially, in Figure 11c, in the case of the nanocomposite. Although DSC did not show an increase of crystallinity in PHB‒5BC compared to pristine PHB, being a bulk analysis, the surface morphology of the nanocomposite showed a higher degree of order and better organization. Although spherulites may not be seen at this magnitude, the twisted lamellar morphology of PHB was visible in Figure 11c [77]. This may be an influence of BC nanofibers. Previous works have shown that cellulose nanofibers, regardless the source or fabrication, act as nucleating agents, enhancing the crystallization and the degree of order in PHB [8,70,76]. Plasma treatment induced visible changes in the surface morphology. The crystalline organization was less visible as compared to untreated samples, similar to SEM observations. The analysis of topographic images (Figure S6) reveals new details. The crystalline lamellar structure is also visible after the plasma treatment; however, the structure is more regular and the surface roughness is lower. Thus, root mean square roughness (RRMS) was determined using the AFM topographic images. RRMS of PHB surface was 19.0 ± 2.3 nm before and 16.6 ± 0.5 nm after the plasma treatment; for the nanocomposite, RRMS was 16.1 ± 1.9 nm before and 13.3 ± 1.1 nm after the plasma treatment. This decrease of surface roughness may result from the high energy and active species, which allow a higher mobility of the PHB chains in plasma-treated samples. Similar observations were reported for PHB films treated with oxygen and carbon dioxide plasma in a vacuum chamber [51].

PHB nanocomposite with 2 wt % bacterial cellulose nanofibers, which led to the best thermal and mechanical properties, was thoroughly investigated by SEM-EDX before and after plasma treatments, with and without ZnO deposition. EDX results (Figure S6 and Table S2) show the presence of less than 1% Si in all the samples and traces of different other elements (Na, K, Mg, Al), which suggests an aluminosilicate filler. Therefore, the particles observed in the SEM images from Figure 10 may be associated to aluminosilicate filler or nucleating agent. The EDX results for ZnO plasma-treated PHB‒2BC clearly show the presence of about 10% ZnO on the surface of nanocomposite. Moreover, the O/C weight ratio is 0.60, so almost identical to the theoretical value for PHB, and slightly decreases after the plasma treatment, from 0.60 to 0.57; however, it strongly increases (to 1.16) after ZnO deposition due to the O atoms in ZnO.

**Figure 12:**
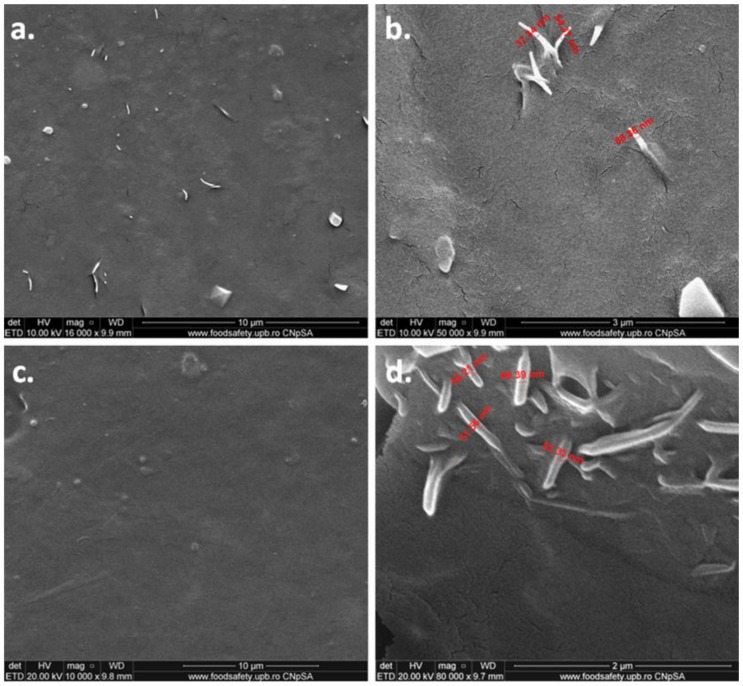
SEM images of PHB‒2BC nanocomposite before (**a**,**b**) and after (**c**,**d**) plasma treatment.

The SEM images of the PHB‒2BC nanocomposite film before and after the plasma treatment are shown in Figure 12. Bacterial cellulose nanofibers are well dispersed on the surface of nanocomposite film and embedded into the PHB matrix. A couple of nanofibers with the top part outside (Figure 12b) were used to determine their size. Nanofibers have a thickness between 30 and 70 nm. After the plasma treatment the surface seems smoother (Figure 12c) and the nanofiber size is in the same range (Figure 12d).

**Figure 13:**
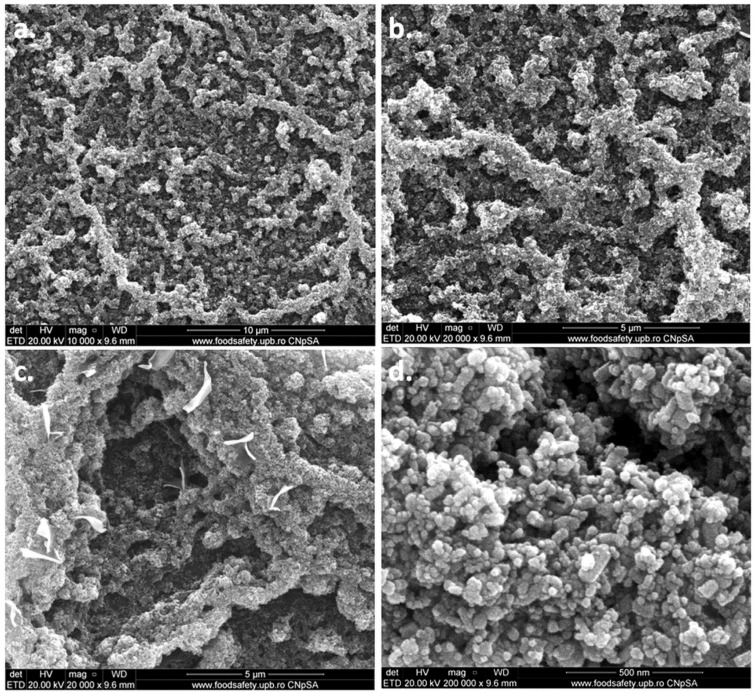
SEM images of PHB‒2BC nanocomposite after ZnO plasma coating.

SEM images of PHB‒2BC nanocomposite after ZnO plasma coating are shown in Figure 13. The ZnO nanoparticles continuously cover the surface of PHB‒2BC nanocomposite; however, they self-aggregate similar to a coral reef structure (Figure 13a,b). This pattern results from the conjugated influence of ZnO and polymeric substrate. SEM image of PHB film right after the treatment (Figure S5) shows a “egg box” structure. We may presume that the holes of this structure will allow the deposition of more ZnO nanoparticles, which will aggregate, forming the coral reef pattern. The plasma coating process also causes the detachment of cellulose fibers and polymer strips, as observed in Figure 13c. Figure 13d shows the aggregated ZnO nanoparticles with a granular shape and an average diameter of only 18 nm. Similar shape and size of ZnO nanoparticles were observed by SEM on the surface of paper impregnated with an ammoniacal suspension of ZnO [78].

### 3.7. Antibacterial Properties 

The antibacterial activity of ZnO was demonstrated by previous works [32,33,36]; however, the influence of a simple Ar plasma exposure on the antibacterial activity of PHAs had not yet been studied.

**Figure 14:**
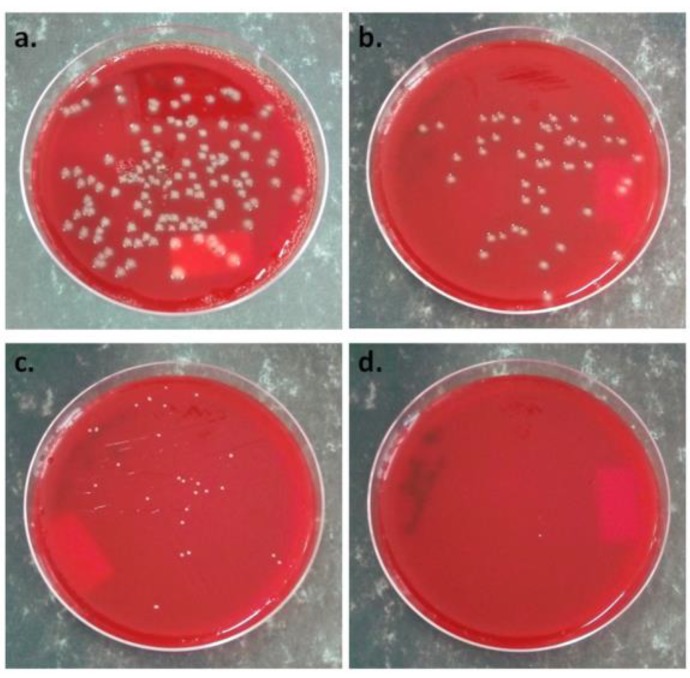
Antibacterial activity of PHB‒2BC nanocomposite films against *Escherichia coli* before (**a**) and after the plasma treatment (**b**) and against *Staphylococcus aureus* before (**c**) and after ZnO plasma coating (**d**).

Previous work has shown better adhesion and proliferation of mouse adipose-derived stem cells on the surface of plasma-treated PHBV film [39]. Moreover, the presence of cellulose in the nanocomposite favors cells proliferation [14,71]. Better compatibility with L929 cells was reported for poly(3-hydroxyoctanoate) (PHO) reinforced with 3 wt % BC compared to pristine PHO due to a more hydrophilic surface [71], similarly for PHB modified with a polyhydroxyalkanoate and 2 wt % BC compared to PHB [14]. Therefore, the number of colonies obtained after incubation of the bacterial suspensions in contact with the untreated and plasma-treated PHB‒2BC nanocomposite films was determined. A decrease by 44% of the *Staphylococcus aureus* bacteria colonies’ mean number, from 100 to 56, was obtained after the plasma treatment. Similarly, 63% inhibition of *Escherichia coli* growth after 24 h was obtained in the case of plasma-treated PHB‒2BC compared to the untreated film (Figure 14a,b). For comparison, a PHB‒2BC‒ZnO nanocomposite was characterized in the same conditions and almost complete inhibition of *Staphylococcus aureus* growth was obtained (Figure 14c,d). This significant decrease of the number of colonies, by 44% in the case of *Staphylococcus aureus* and 63% in the case of *Escherichia coli*, shows that the plasma treatment is able to induce antibacterial activity. Similar antibacterial efficiency against *Escherichia coli* was reported in the case of a PHB blended with a quaternarized N-halamine polymer obtained using a complicate chemical route [79]. Besides biodegradability and eco-friendliness, the new nanocomposite films show good thermal and mechanical properties and increased antibacterial activity. Thus, plasma-treated PHB‒2BC nanocomposite films are proposed for food packaging to protect fresh foods from decay.

## 4. Conclusions

Biodegradable and eco-friendly films of poly(3-hydroxybutyrate) modified with bacterial cellulose nanofibers were prepared using a melt compounding‒compression molding technique and surface-treated by a plasma or ZnO nanoparticle plasma coating. The thermal, mechanical and antibacterial properties of these films were studied for possible applications in food packaging. The thermal stability, crystallinity and melting behavior of nanocomposites were not changed by the plasma treatment; however, a remarkable increase in stiffness and strength was obtained. ZnO plasma coating consisted of a continuous layer of self-aggregated ZnO nanoparticles and led to the improvement of the crystallinity and mechanical properties of the PHB‒2BC nanocomposite. Higher antibacterial activity was noted after the plasma treatment and complete inhibition of *Staphylococcus aureus* growth after ZnO nanoparticle plasma coating. Plasma-treated PHB nanocomposites containing bacterial cellulose nanofibers are proposed as a “green” solution for food packaging.

## Figures and Tables

**Table 1:** TGA results for PHB nanocomposites before and after the plasma treatment.

Nanocomposites	*T*_5%_ (°C)	*T*_on_ (°C)	*T*_max_ (°C)	*R*_600_ (%)
PHB	191.9	254.1	266.3	2.23
PHBp	202.4	255.5	270.7	2.26
PHB‒1BC	195.3	255.2	268.4	2.22
PHB‒1BCp	196.7	256.6	269.9	2.29
PHB‒2BC	192.4	256.4	267.9	2.29
PHB‒2BCp	203.0	259.1	270.5	2.43
PHB‒5BC	192.8	255.4	266.7	2.48
PHB‒5BCp	194.4	247.7	261.5	2.50
PHB‒2BC‒ZnO	183.5	251.0	265.0	3.43

**Table 2:** Data obtained from the analysis of DSC melting and cooling thermograms.

Nanocomposites	*T*_m1_/*T*_m2_ °C	Δ*H*_m_ J/g	Δ*H*_m1_/Δ*H*_m2_ J/g	*T_c_* °C	Δ*H_c_* J/g	C* %
PHB	163.8/170.5	76.1	57.7/18.4	112.2	73.6	57.9
PHBp	163.0/170.7	75.5	53.2/22.3	111.8	73.8	57.5
PHB‒0.5BC	164.2/169.0	75.1	63.1/12.0	114.8	75.2	57.4
PHB‒0.5BCp	163.8/170.4	74.6	55.3/19.3	116.5	74.6	57.1
PHB‒1BC	163.6/170.5	75.8	61.3/14.5	114.1	73.7	58.3
PHB‒1BCp	162.9/170.8	74.2	50.5/23.7	115.2	74.0	57.0
PHB‒2BC	163.6/170.5	74.7	56.5/18.2	114.6	73.5	58.0
PHB‒2BCp	162.8/170.6	72.4	50.7/21.7	115.1	72.1	56.2
PHB‒5BC	164.4/169.0	71.2	60.3/10.9	114.1	69.8	57.0
PHB‒5BCp	163.0/170.8	71.5	49.7/21.8	114.0	71.0	57.3
PHB‒2BC‒ZnO	162.7/170.6	76.5	51.7/24.8	113.7	77.5	59.4

* calculated with Equation (1); the amount of TBC plasticizer (10 wt %) was extracted from the *w*_PHB_ value.

*T*_m1_/*T*_m2_—melting temperature of PHB corresponding to the double melting peaks; *T*_c_—crystallization temperature; Δ*H*_m1_/Δ*H*_m2_—melting enthalpy of PHB corresponding to the double melting peaks; Δ*H*_m_—total melting enthalpy.

**Table 3:** DMA results: glass transition of PHB (*T*_g_), T_α_* transition (crystal‒crystal slip) and storage modulus (*E’*) values at different temperatures.

Nanocomposites	*T*_g_ [°C]	*T*_α_* [°C]	*E’* [MPa] −15 °C	*E’* [MPa] 30 °C	*E’* [MPa] 100 °C
PHB	14.2	123	3807	2687	1024
PHBp	16.2	126	3833	2827	1141
PHB‒2BC	17.1	122	3456	2532	901
PHB‒2BCp	18.0	125	3980	3000	1290
PHB‒5BC	14.9	125	3652	2768	1075
PHB‒5BCp	17.8	128	4289	3324	1058
PHB‒2BC‒ZnO	15.5	136	3950	3064	1311

## Supplementary Materials

The following are available online at http://www.mdpi.com/2073-4360/10/11/1249/s1, Figure S1: DTG curves (300–350 °C) of PHB nanocomposites before and after the plasma treatment; Figure S2: FTIR spectra of PHB and PHB nanocomposites with 2 wt % and 5 wt % BC; Figure S3: FTIR spectra of PHB (A), PHB‒2BC (B) and PHB‒5BC (C) before and after the plasma treatments; Figure S4: DSC first melting and cooling scans for ZnO plasma-coated PHB‒2BC compared to the untreated nanocomposite; Figure S5: SEM image of PHB right after the plasma treatment; Figure S6: AFM topographic images of PHB and PHB‒5BC before (a,c) and after the plasma treatment (b,d); Figure S7: EDX results for PHB‒2BC and PHB‒2BC‒Zn; Table S1: EDX data for PHB‒2BC nanocomposite before and after plasma and ZnO plasma coating; Table S1: Peak assignments in the FTIR spectra of PHB and nanocomposites in the 3050–2800 cm^−1^ region.
