# Supplementary material for: Poly(3-hydroxybutyrate) Modified by Nanocellulose and Plasma Treatment for Packaging Applications
Source: Polymers (Basel). 2018 Nov 11;10(11):1249. doi: 10.3390/polym10111249 (PMC6401738; doi:10.3390/polym10111249)
Supplement: Supplementary file 1 [file polymers-10-01249-s001.pdf]

Supplementary Materials

Poly(3-hydroxybutyrate) Modified by Nanocellulose and Plasma Treatment for Packaging Applications

Denis Mihaela Panaitescu,[1,*] Eusebiu Rosini Ionita,[2] Cristian Andi Nicolae,[1] Augusta Raluca Gabor,[1] Maria Daniela Ionita,[2] Roxana Trusca,[3] Brindusa-Elena Lixandru,[4] Irina Codita,[4,5] Gheorghe Dinescu[2]

[1]National Institute for Research & Development in Chemistry and Petrochemistry - ICECHIM, Polymer Department, 202 Spl. Independentei, 060021, ca_nicolae@yahoo.com (C.A.N.); ralucagabor@yahoo.com (A.R.G.);

[2]National Institute for Laser, Plasma and Radiation Physics, Atomistilor 409, Magurele-Bucharest, 077125 Ilfov, Romania; ionita.rosini@infim.ro (E.R.I.); daniela.ionita@infim.ro (M.D.I.); dinescug@infim.ro (G.D.)

[3]Science and Engineering of Oxide Materials and Nanomaterials, University Politehnica of Bucharest, 1-7 Gh. Polizu Street, 011061 Bucharest, Romania; truscaroxana@yahoo.com (R.T.)

[4]"Cantacuzino" National Medical-Military Institute for Research and Development, 103 Spl. Independentei, 050096, Bucharest, Romania, brandusa_lixandru@yahoo.com (B.E.L); icodita@cantacuzino.ro (I.C.)

[5]Carol Davila University of Medicine and Pharmacy, Bulevardul Eroii Sanitari 8, 050474, Bucharest, Romania

*Correspondence: panaitescu@icechim.ro; Tel.: +004 0213163068

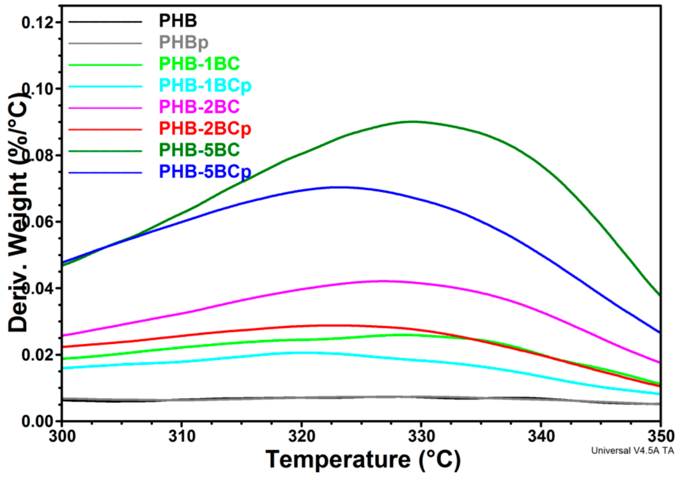

Figure S1. DTG curves (300–350 °C) of PHB nanocomposites before and after the plasma treatment.

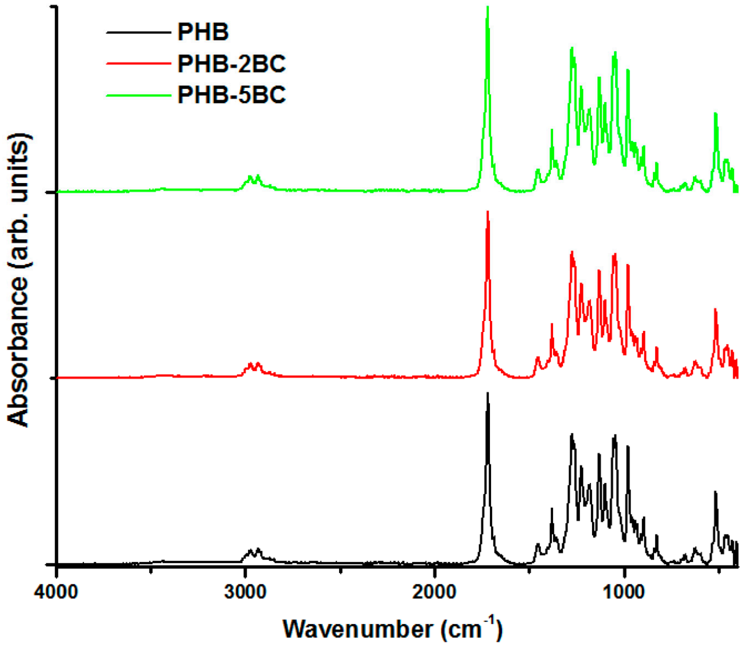

Figure S2. FTIR spectra of PHB and PHB nanocomposites with 2 wt % and 5 wt % BC.

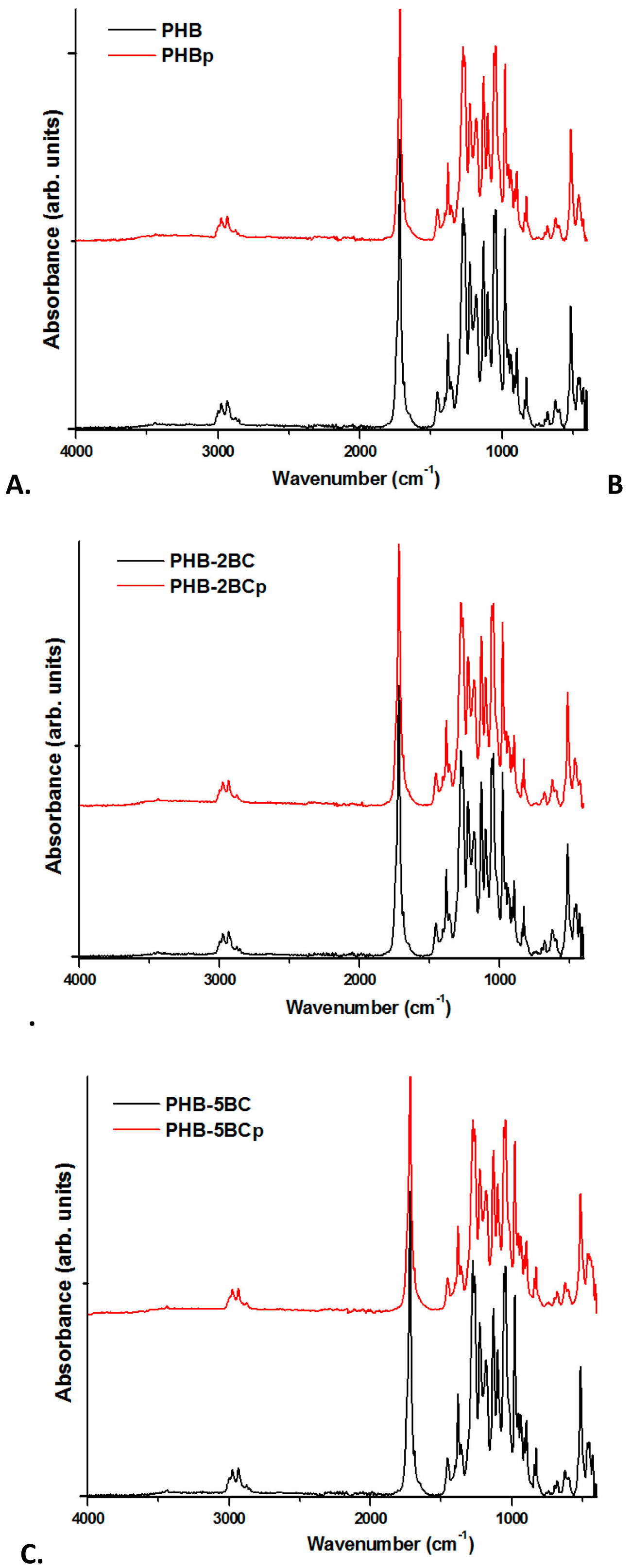

Figure S3. FTIR spectra of PHB (**A**), PHB–2BC (**B**) and PHB–5BC (**C**) before and after the plasma treatments.

Table S1. Peak assignments in the FTIR spectra of PHB and nanocomposites in the 3050–2800 cm^{-1} region.

Wavenumber (cm^{-1})	Assignment	References
3007	CH_3 asymmetric stretching vibrations, indicating the presence of intermolecular CH…O hydrogen bonds	Zhang J.; Sato, H.; Noda, I.; Ozaki, Y. *Macromolecules* 2005, 38, 4274-4281
2997	CH_3 asymmetric stretching vibrations	Sato, H.; Murakami, R.; Padermshoke, A.; Hirose, F.; Senda, K.; Noda, I.; Ozaki, Y. *Macromolecules* 2004, 37, 7203-7213; Zhang J.; Sato, H.; Noda, I.; Ozaki, Y. *Macromolecules* 2005, 38, 4274-4281; Padermshoke, A.; Katsumoto, Y.; Sato, H.; Ekgasit, S.; Noda, I.; Ozaki, Y. *Spectrochim. Acta A* 2005, 61, 541–550.
2976/2968	CH_3 asymmetric stretching vibrations; the pear of bands resulting from the crystal field splitting, caused by inter- or intramolecular interactions	Sato, H.; Murakami, R.; Padermshoke, A.; Hirose, F.; Senda, K.; Noda, I.; Ozaki, Y. *Macromolecules* 2004, 37, 7203-7213; Zhang J.; Sato, H.; Noda, I.; Ozaki, Y. *Macromolecules* 2005, 38, 4274-4281; Padermshoke, A.; Katsumoto, Y.; Sato, H.; Ekgasit, S.; Noda, I.; Ozaki, Y. *Spectrochim. Acta A* 2005, 61, 541–550.
2934/2923	CH_2 asymmetric stretching vibrations; the pear of bands resulting from the crystal field splitting, caused by inter- or intramolecular interactions	Zhang J.; Sato, H.; Noda, I.; Ozaki, Y. *Macromolecules* 2005, 38, 4274-4281; Padermshoke, A.; Katsumoto, Y.; Sato, H.; Ekgasit, S.; Noda, I.; Ozaki, Y. *Spectrochim. Acta A* 2005, 61, 541–550.
2874	CH_3 symmetric stretching vibrations	Sato, H.; Murakami, R.; Padermshoke, A.; Hirose, F.; Senda, K.; Noda, I.; Ozaki, Y. *Macromolecules* 2004, 37, 7203-7213; Socrates, G. *Infrared and Raman characteristic group frequencies*, 2001, pp. 50–67; Zhang J.; Sato, H.; Noda, I.; Ozaki, Y. *Macromolecules* 2005, 38, 4274-4281.
2851	CH_2 symmetric stretching vibrations	Socrates, G. *Infrared and Raman characteristic group frequencies*, 2001, pp. 50–67.

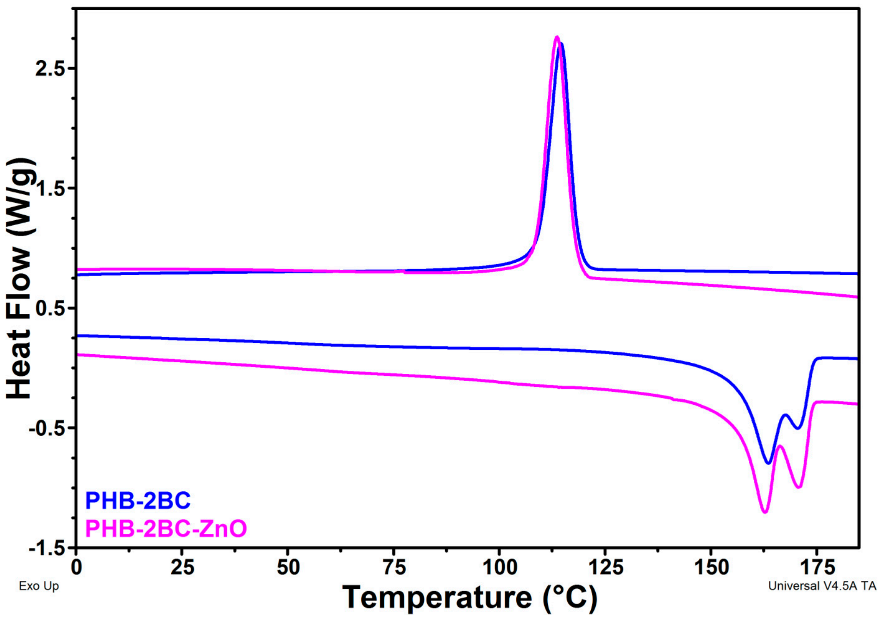

Figure S4. DSC first melting and cooling scans for ZnO plasma-coated PHB–2BC compared to the untreated nanocomposite.

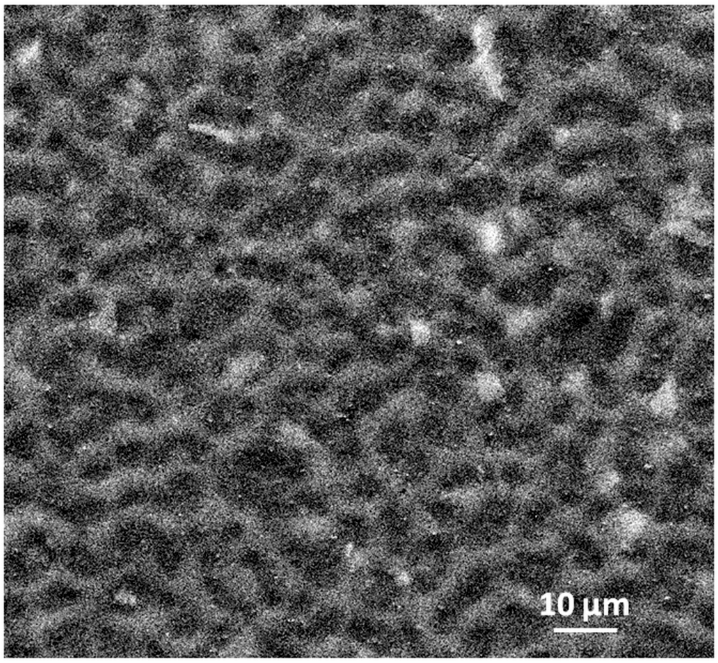

Figure S5. SEM image of PHB right after the plasma treatment.

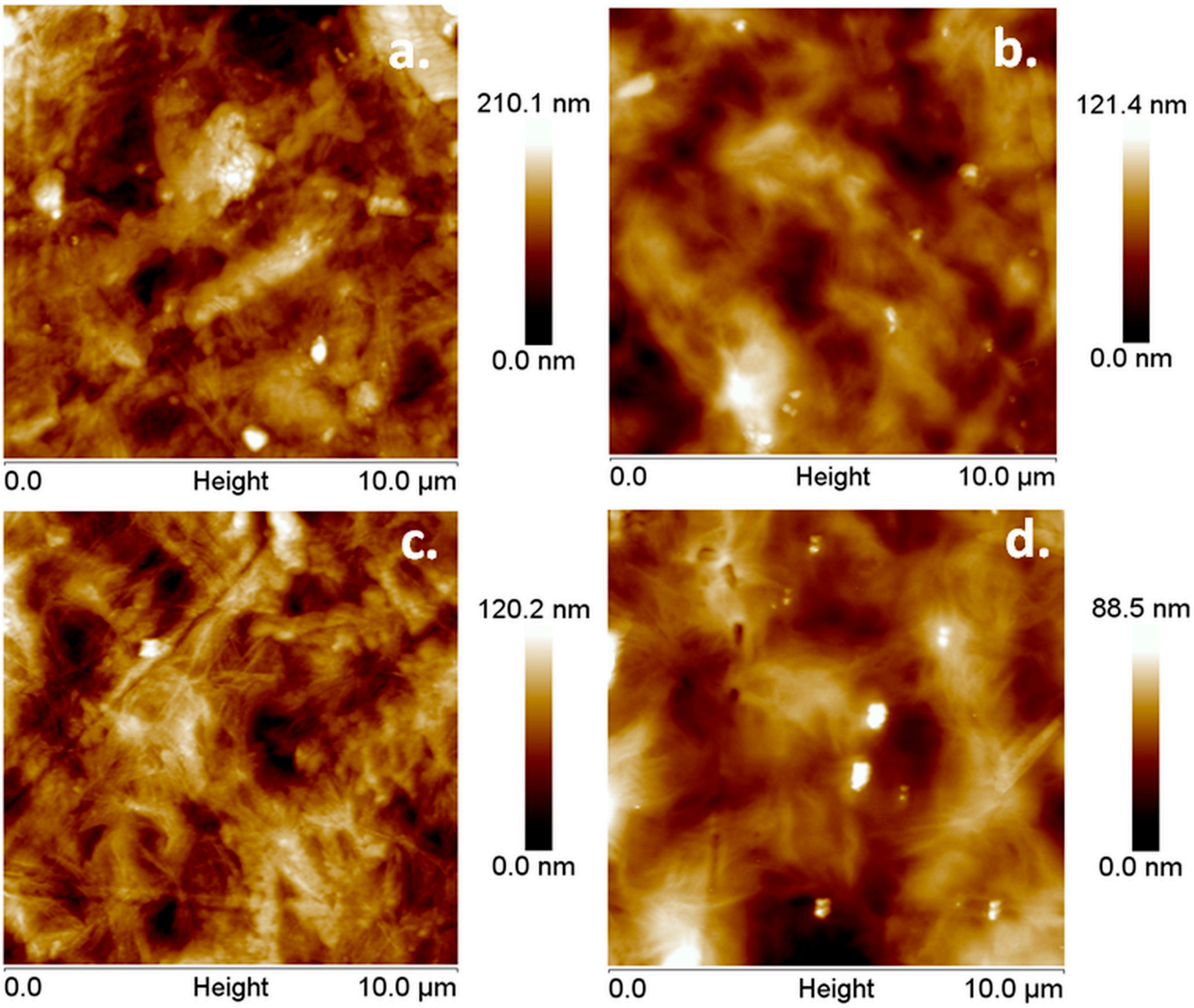

Figure S6. AFM topographic images of PHB and PHB–5BC before (**a**,**c**) and after the plasma treatment (**b**,**d**).

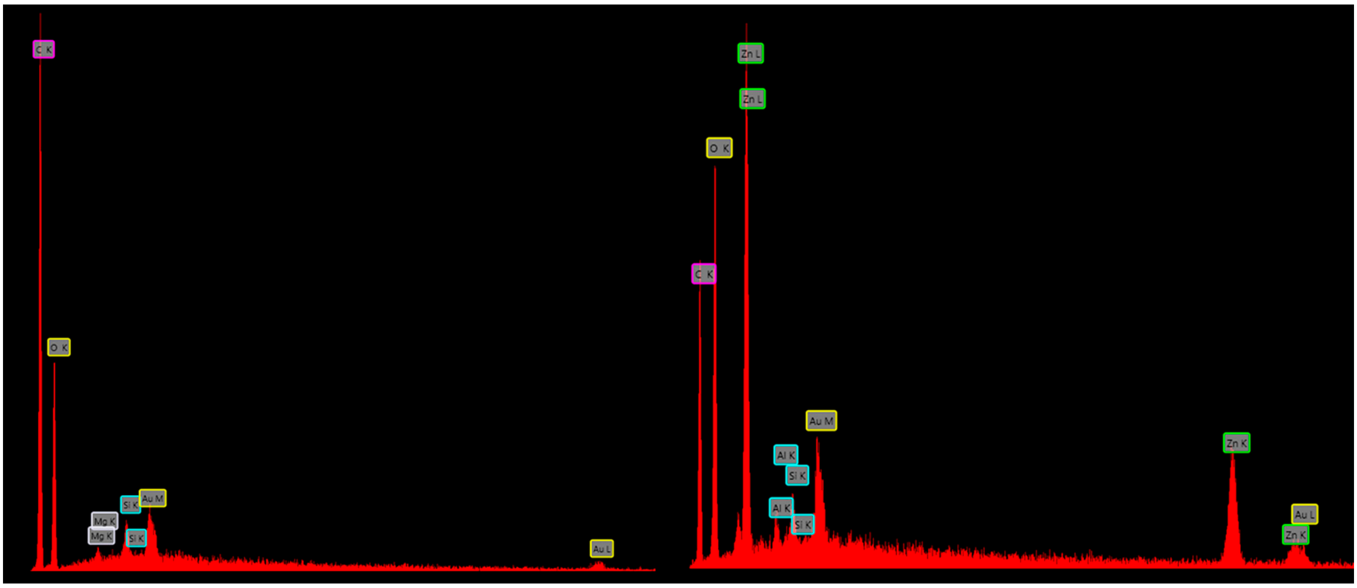

Figure S7. EDX results for PHB–2BC (left) and PHB–2BC–Zn (right). .

Table S2. EDX data for PHB–2BC nanocomposite before and after plasma and ZnO plasma coating.

PHB nanocomposite—different treatments	Elemental composition (weight %)*							
	C	O	Si	Zn	Mg	Na	K	Al
PHB–2BC	58.5	38.6	0.6	-	0.3	-	-	-
PHB–2BCp	60.7	34.5	0.5	-	0.3	1.8	0.2	-
PHB–2BC–ZnO	41.1	47.6	0.8	9.9	-	-	-	0.5

*the rest up to 100% is Au element due to the sputter-coating of the film surface before the SEM-EDX measurement.
